# Body mass index as a key determinant of oxidative stress in women undergoing infertility treatment

**DOI:** 10.3389/frph.2026.1790127

**Published:** 2026-04-30

**Authors:** Katarzyna Olszak-Wąsik, Andrzej Tukiendorf, Rafał Kurzawa, Adnan Karaibrahimoğlu, Aleksandra Kasperczyk

**Affiliations:** 1 Department of Gynaecology, Obstetrics and Oncological Gynaecology, Faculty of Medical Sciences in Zabrze, Medical University of Silesia, Zabrze, Poland; 2 TFP Fertility Macierzyństwo Kraków, Krak&oacute;w, Poland; 3 TFP Fertility Vitrolive, Szczecin, Poland; 4 Department of Physical Education and Sport, Faculty of Physical Education and Physiotherapy, Opole University of Technology, Opole, Poland; 5 Department of Gynecology and Reproductive Health, Pomeranian Medical University, Szczecin, Poland; 6 Faculty of Medicine, Biostatistics and Medical Informatics Department, Süleyman Demirel Üniversitesi, Isparta, Türkiye; 7 Department of Biochemistry, Faculty of Medical Sciences in Zabrze, Medical University of Silesia, Zabrze, Poland

**Keywords:** infertility, *in vitro* fertilization, oxidative stress, taxonomy-based analysis, BMI

## Abstract

**Introduction:**

Infertility treatment may be influenced by systemic metabolic and redox imbalances. This prospective observational (cross-sectional) study aimed to evaluate the impact of clinical risk factors on oxidative stress markers and components of the antioxidant defence system in women undergoing *in vitro* fertilization.

**Methods:**

To capture complex redox interactions, we applied a triad-based analytical framework to assess relationships between clinical variables and oxidative stress–related biomarkers.

**Results:**

The results indicate that body mass index (BMI) is the strongest determinant of redox imbalance among the analyzed risk factors. Increasing BMI, particularly in the overweight and obese ranges, was associated with a shift toward a pro-oxidative state, as reflected by reduced superoxide dismutase (SOD) activity, alterations in SOD isoenzyme activity, increased lipid peroxidation, and decreased total antioxidant capacity. None of the other evaluated risk factors demonstrated similarly consistent associations with the oxidative stress biomarker panel.

**Discussion:**

The applied statistical modeling approach enabled the identification of potential cause–and–effect relationships between infertility risk factors and oxidative stress–related biomarkers, identifying BMI as a key modifiable determinant of redox homeostasis, with antioxidant enzymes playing a central role.

## 1 Introduction

Female reproductive function is highly sensitive to systemic metabolic disturbances that can disrupt hormonal regulation, cellular homeostasis, and reproductive physiology (1). In assisted reproductive technologies, including *in vitro* fertilization (IVF), clinical and biochemical responses observed in patients may be influenced by several categories of risk factors, including the etiology of infertility, patient characteristics, and systemic metabolic status. These determinants may influence systemic oxidative–antioxidant balance and inflammatory activity, which can be reflected in circulating biomarkers, including total antioxidant capacity, oxidant status, lipid peroxidation products, antioxidant enzymes, and redox-sensitive protein components measured in serum (2).

Ovarian factor infertility may be associated with disturbances in systemic antioxidant defense and protein metabolism. Altered ovarian function has been linked with changes in oxidative stress parameters and antioxidant enzyme activity (3)

In contrast, tubal factor infertility primarily reflects a mechanical impairment of gamete transport rather than a systemic metabolic disturbance. Consequently, its association with circulating oxidative stress markers is generally considered indirect and limited. For this reason, women with tubal infertility are often used as reference groups in studies evaluating oxidative and antioxidant biomarkers in reproductive medicine Ambildhuke et al. (4).

Similarly, idiopathic infertility represents a heterogeneous clinical category in which the underlying mechanism remains unclear. In this context, systemic oxidative stress markers, including lipid peroxidation products or antioxidant enzymes, have been proposed as potential biomarkers that may reflect subtle metabolic or inflammatory disturbances not captured by conventional clinical diagnostics (5).

A more pronounced relationship with oxidative processes has been described in endometriosis, a condition characterized by chronic inflammation and increased oxidative stress. Patients with endometriosis have been shown to exhibit elevated markers of lipid peroxidation, such as malondialdehyde (MDA) and lipid hydroperoxides (LPH), together with alterations in antioxidant defense systems, including SOD activity. These findings support the concept that oxidative stress contributes to the pathophysiology of endometriosis-related infertility (6).

In cases of isolated male factor infertility, the primary pathological mechanism is generally attributed to impaired sperm parameters rather than a defined female reproductive disorder. Therefore, when evaluating serum biomarkers in women undergoing IVF, male factor infertility is generally not expected to represent a major determinant of the systemic oxidative or inflammatory profile of the female partner (7).

Beyond infertility etiology, patient-related characteristics may also influence systemic oxidative balance. Advancing age has been associated with increased oxidative stress and altered antioxidant enzyme activity, which may manifest as changes in serum markers such as lipid peroxidation products and antioxidant enzymes. An age-related biological determinant is ovarian reserve, commonly assessed using anti-Müllerian hormone (AMH). Although AMH primarily reflects the quantity of remaining ovarian follicles, it may indirectly correspond with metabolic and oxidative status, as diminished ovarian reserve has been associated with increased oxidative stress and impaired cellular defense mechanisms in reproductive tissues (8, 9).

Among the factors influencing female reproductive health, body mass index (BMI) represents an important and potentially modifiable determinant. Increasing evidence indicates that deviations from the normal BMI range are associated with metabolic and endocrine disturbances that may adversely affect reproductive function (1). BMI, calculated as body weight in kilograms divided by the square of height in meters (kg/m^2^), is widely used to classify individuals as underweight (<18.5 kg/m^2^), normal weight (18.5–24.9 kg/m^2^), overweight (25.0–29.9 kg/m^2^), or obese (≥30.0 kg/m^2^) (10).

Overweight and obesity are associated with chronic low-grade inflammation and increased generation of reactive oxygen species, leading to disturbances across multiple oxidative and antioxidant pathways (11).

The antioxidant defence system comprises both enzymatic and non-enzymatic components that protect cells against oxidative damage. Among them, superoxide dismutases (SODs) constitute the primary endogenous enzymatic defence against reactive oxygen species (ROS) by catalyzing the conversion of superoxide radicals into less reactive molecules (12) Steinmetz et al. (13). Mammalian cells contain three major SOD isoforms: the cytosolic copper–zinc form (SOD1; Cu/ZnSOD), the mitochondrial manganese form (SOD2; MnSOD), and the extracellular form (SOD3; EC-SOD). Among these, SOD1 accounts for approximately 50%–80% of total SOD activity, making alterations in its function particularly relevant for maintaining cellular redox balance (14).

Beyond SOD, cellular redox balance is supported by a broader network of enzymatic and non-enzymatic antioxidant systems together with oxidative stress–related biomarkers, including catalase, ceruloplasmin, malondialdehyde, lipofuscin, and total antioxidant capacity.

Catalase (CAT) is a tetrameric antioxidant enzyme predominantly localized in peroxisomes, where it plays a key role in cellular redox homeostasis by decomposing hydrogen peroxide into water and oxygen (15). Dysregulation of catalase activity has been associated with the development and progression of various disorders (16). By limiting hydrogen peroxide accumulation, catalase reduces oxidative stress and lipid peroxidation while supporting the activity of other antioxidant systems, including superoxide dismutase, thereby contributing to the maintenance of cellular redox balance and immune homeostasis (17).

Ceruloplasmin (CP) is a non-enzymatic antioxidant that contributes to antioxidant defence through its ferroxidase and related oxidase activities, enabling the conversion of Fe^2^⁺ to the less reactive Fe^3^⁺ without generating additional reactive oxygen species. By limiting the availability of ferrous iron, CP helps prevent Fenton-type reactions and protects tissues from oxidative injury. In addition, ceruloplasmin may influence nitric oxide–related pathways and contribute to the maintenance of cellular redox balance (18).

Total antioxidant capacity (TAC) reflects the overall antioxidant potential of biological systems by integrating the combined activity of enzymatic and non-enzymatic antioxidants, including superoxide dismutase, catalase, glutathione peroxidase, and low-molecular-weight antioxidants such as vitamins C and E. Measurement of TAC in biological fluids provides a useful indicator of the global antioxidant status of the organism (19).

Malondialdehyde (MDA), together with SOD, serves as an *in vivo* indicator of oxidative stress (20). MDA is a secondary lipid peroxidation product generated through enzymatic and non-enzymatic degradation of arachidonic acid and other polyunsaturated fatty acids. Compared with reactive oxygen species, MDA is chemically more stable and membrane-permeable, which facilitates its accumulation and biological activity (21).

Lipofuscin (LPS) is an endogenous marker of chronic oxidative stress formed through incomplete degradation of lipid peroxidation products and oxidatively modified proteins. It accumulates primarily within lysosomes of long-lived cells, with levels increasing alongside the intensity and duration of oxidative stress, making it a useful indicator of cumulative oxidative damage. Recent studies suggest that lipofuscin may also serve as a marker of oxidative stress in reproductive tissues, including the ovary, where it may indirectly reflect oocyte quality and ovarian reserve (22, 23).

Together, these oxidative stress–related molecules reflect different aspects of cellular redox balance and provide a useful conceptual framework for exploring the relationship between metabolic disturbances and oxidative stress in women undergoing infertility treatment.

## 2 Materials and methods

This prospective observational (cross-sectional) study we aimed to investigate which infertility risk factors—and through which mechanisms—modify oxidative stress markers and components of the antioxidant defence system. A total of 77 women undergoing IVF treatment for infertility were enrolled in the study. All participants were recruited at TFP Fertility, Vitrolive, Poland. The characteristics of the study population are presented in Table 1. Ovarian stimulation was performed using gonadotropin preparations within either antagonist or agonist protocols. In the antagonist protocol, ganirelix (0.25 mg/day) was administered to prevent a premature luteinizing hormone surge. In the agonist protocol, triptorelin was initiated on day 20 of the preceding menstrual cycle to achieve pituitary downregulation. Ovarian stimulation was carried out using recombinant follitropin alfa or menotropin, with starting doses individualized according to ovarian reserve markers, particularly anti-Müllerian hormone (AMH) levels. The administered gonadotropin doses ranged from 150 to 375 IU per day, depending on the patient's ovarian reserve and clinical response. Both stimulation protocols are routinely used in clinical IVF practice and were selected according to individual clinical indications. Final oocyte maturation was triggered with either recombinant human chorionic gonadotropin (two ampoules of 6,500 IU each) or triptorelin 0.2 mg.

**Table 1:** Clinical characteristics of patient risk factors and clinical responses.

Risk factor	Status/value	*N*	%
Ovarian factor	no/yes	57/18	76/24
Tubal factor	no/yes	64/11	85.3/14.7
Idiopathic factor	no/yes	53/22	70.7/29.3
Endometriosis	no/yes	69/5	93.2/6.8
Male factor	no/yes	38/39	49.4/50.6
IVF attempt number:	First IVF	54	70.1
	Up to 2 previous IVF attempts	17	22.1
	More than 2 previous IVF attempts	6	7.8
Risk factor	n	mean ± SD, median	range
Age	77	33.3 ± 4.3, 34.0	23.0–44.0
Number of hMG/FSH administration days	77	9.6 ± 2.0, 9.0	4.0–14.0
AMH (anti-Müllerian hormone)	76	3.47 ± 2.93, 2.72	0.40–16.81
BMI (body mass index)	74	22.7 ± 3.5, 21.8	17.4–36.3
Clinical response	n	mean ± SD, median	range
protein	77	70.2 ± 7.14, 70.0	52.1–114.8
Sulfhydryl (SH) groups	77	319 ± 61, 312	193–754
Protein-bound sulfhydryl groups	77	4.56 ± 0.90, 4.50	2.70–11.31
ceruloplasmin (CER)	77	35.2 ± 9.8, 33.4	16.8–69.9
Total antioxidant capacity (TAC)	77	1.14 ± 0.08, 1.13	0.92–1.29
Total oxidant status (TOS)	77	11.1 ± 5.1, 10.1	3.6–47.4
Lipid hydroperoxides (LPH)	77	6.971 ± 2.84, 6.59	2.34–25.30
Superoxide dismutase (SOD)	77	20.0 ± 1.3, 20.1	16.8–22.9
Manganese superoxide dismutase (MnSOD)	77	10.4 ± 0.9, 10.5	8.2–12.8
Copper-zinc superoxide dismutase (CuZnSOD)	77	9.62 ± 1.30, 9.61	5.64–12.43
Lipofuscin (LPS)	77	328 ± 94, 318	168–673
Malondialdehyde (MDA)	77	1.00 ± 0.31, 1.01	0.50–2.09

Serum oxidative stress and antioxidant system markers were analyzed in samples obtained from 77 patients at the Department of Biochemistry, Silesian Medical University, School of Medicine, and the Division of Dentistry in Zabrze, Poland.

The study was approved by the Bioethics Committee of the Regional Medical Chamber in Szczecin, Poland (approval no. 12/KB/VI/2016). Written informed consent was obtained from all participants prior to inclusion in the study and sample collection.

### 2.1 Biochemical analyses

Venous blood samples were obtained on the day of ovulation triggering. After clotting and centrifugation, serum was separated and aliquoted into cryovials and stored in liquid nitrogen until further analysis.

Serum was analyzed for a panel of oxidative stress biomarkers, encompassing both enzymatic and non-enzymatic antioxidants (12 markers in total). The following parameters were measured: protein sulfhydryl (SH) groups, protein-bound sulfhydryl groups, ceruloplasmin (CER), total antioxidant capacity (TAC), total oxidant status (TOS), lipid hydroperoxides (LPH), total superoxide dismutase (SOD), manganese superoxide dismutase (MnSOD), copper–zinc superoxide dismutase (CuZnSOD), lipofuscin (LPS), and malondialdehyde (MDA).

Total serum protein concentration was quantified using an A25 biochemistry analyzer (BioSystems S.A., Barcelona, Spain) according to the manufacturer's protocol.

#### 2.1.1 Antioxidant enzymes

The activity of superoxide dismutase (SOD) and its isoenzymes—manganese SOD (MnSOD) and copper–zinc SOD (CuZnSOD)—was determined using the method of Oyanagui (24). One nitric unit (NU) corresponds to a 50% inhibition of nitric ion formation. Activities were reported as NU/mL. Catalase (CAT) activity was assessed using the Johansson and Borg method (25). The assay measures formaldehyde produced from the reaction between methanol and H₂O₂ in the presence of CAT; absorbance of the purpald–formaldehyde complex was recorded at 550 nm. Results were expressed as U/g protein.

#### 2.1.2 Nonenzymatic antioxidants

Total antioxidant capacity (TAC) was quantified according to Erel (26). This colorimetric assay measures the capacity of antioxidants to suppress the formation of ABTS⁺ radicals [2,2′-azinobis(3-ethylbenzothiazoline)-6-sulfonate]. The decrease in absorbance at 660 nm indicates antioxidant activity. Analyses were performed using an automated PerkinElmer analyzer calibrated with Trolox (Sigma-Aldrich), and results were expressed in mmol/L.

Ceruloplasmin (CER) concentration was assessed according to the method of Richterich (27), with absorbance recorded at 546 nm, and values expressed in mg/dL.

Protein sulfhydryl (SH) groups were measured following the procedure of Koster et al. (28). In this assay, 5,5′-dithiobis (2-nitrobenzoic acid) (DTNB) is reduced by thiol-containing compounds, producing the yellow 5-thio-2-nitrobenzoate anion, which is detected at 412 nm. Measurements were performed using an automated PerkinElmer analyzer (USA).

#### 2.1.3 Markers of oxidative stress

Total oxidant status (TOS) was evaluated using Erel's method (29), in which oxidants present in the sample oxidize ferrous ions into ferric ions in an acidic medium. The ferric–xylenol orange complex was quantified at 560 nm on a PerkinElmer analyzer, and TOS results were expressed in μmol/L.

Lipofuscin (LPS) was quantified according to Jain (30), with results expressed in relative fluorescence units (RF) corresponding to a fluorescence standard of 0.1 mg/mL quinidine sulfate in 0.1N sulfuric acid.

Malondialdehyde (MDA) was measured using the thiobarbituric acid-reactive substances (TBARS) method of Ohkawa et al. (31). Samples were incubated with sodium dodecyl sulphate, acetic acid, and thiobarbituric acid at 95°C for 1 h, extracted with butanol–pyridine, and the organic phase was read fluorometrically at 552 nm (excitation 515 nm) using a SHIMADZU fluorometer. Results were expressed as MDA equivalents using tetraethoxypropane as the standard (μmol/L).

### 2.2 Statistical analysis

In this study, instead of assessing enzymes/biomarkers individually, we adopted a triad-based structural framework to analyze clinical response variables related to oxidative stress (Y1–Y12, see Table 3 for details). Triads were selected as the fundamental analytical unit because they represent the minimal dimensional structure in which non-trivial geometric relationships can be meaningfully defined and visualized. Given the relatively small sample size, this analytical framework should be interpreted primarily as an exploratory structural approach aimed at identifying potential patterns of association rather than establishing definitive causal relationships. This exploratory framework was designed to identify structural relationships among biomarkers and clinical variables rather than to construct predictive models.

The twelve clinical response variables (Y1–Y12) generate C(12,3) = 220 distinct triads, each representing a unique three-dimensional biochemical space. The triad-based approach was selected specifically to reduce dimensional complexity by analyzing minimal three-variable structures rather than the full multidimensional biomarker space simultaneously. For every triad, a complete 77 × 77 matrix of pairwise taxonomic dissimilarities between patients was constructed. These dissimilarities were quantified using the Marczewski–Steinhaus (M–S) metric, in which the symmetric taxonomic distance (D) between two patients (A and B) is defined as: *D* = |*A–B*|/max(*A,B*), where the numerator represents the absolute difference between A and B, and the denominator is their maximum value Marczewski Edward & Steinhaus Hugo (32).

We used the M–S method because it differs from traditional regression-based approaches, which rely on predefined associations between risk factors and clinical outcomes. As a result, our framework is unsupervised and characterizes the intrinsic geometric organization of the data without imposing linear assumptions. The M–S distance measures relative rather than absolute differences between observations. Unlike Euclidean distance, which aggregates squared deviations across all dimensions, the M–S metric focuses on the proportion of difference relative to the maximum observed value within each pairwise comparison. This makes it inherently scale-invariant after standardization and more suitable when the primary interest lies in structural dissimilarity patterns rather than magnitude alone.

After computing the M–S distance matrices for all 220 triads, each matrix was subjected to hierarchical agglomerative clustering using complete linkage. The resulting dendrograms were then evaluated based on the structure of their top-level split. Specifically, each tree was cut at a height that produced exactly two clusters (*k* = 2). To quantify how evenly the population was partitioned, we defined a balance coefficient as: balance = min (*n*1, *n*2)/(*n*1 + *n*2), where *n*1 and *n*2 denote the number of patients in the two clusters and *n* = *n*1+*n*2 represents the total sample size. This coefficient reflects the symmetry of the top-level division and ranges from 0 (highly unbalanced) to 0.5 (perfectly even split).

Dendrograms were retained only if the two clusters were reasonably balanced according to the predefined threshold 0.30 ≤balance ≤0.70. This requirement ensured that each cluster contained at least 30% of the total number of patients, thereby avoiding trivially small clusters and highlighting triads that produced meaningful structural segregation in the population. In addition, this balance constraint guarantees sufficient sample sizes within each cluster for subsequent statistical comparisons. Through this procedure, latent heterogeneity within the patient population may be explored—patterns of subgroup separation that may remain invisible to conventional regression-based methods—and linked to specific biological determinants (i.e., risk factors X1–X10, see Table 3 for details).

Based on the dendrograms that satisfied the balance criterion, all 77 patients were assigned to either cluster 1 or cluster 2 according to the top-level division of each selected tree. This cluster membership yielded a binary grouping variable reflecting the structural segregation induced by the corresponding triad of clinical response variables (Y1–Y12).

For these two derived clusters, differences in the distributions of the ten risk factors (X1–X10) were quantified. Depending on the statistical properties of each variable, cluster comparisons were performed using either the Wilcoxon rank–sum test or Student's *t*-test. Finally, for each statistically significant result (*p* < 0.05), we identified the individual enzymes/biomarkers within the corresponding triad that were associated with the observed differentiation of the risk factors X1–X10.

## 3 Results

The descriptive statistics of the study population, including analyzed risk factors and clinical response variables, are summarized in Table 1.

Based on the taxonomic analysis of all 220 possible triadic combinations (#1–#220) of the 12 biomarkers (Y1–Y12), 30 triads were selected that met the predefined criterion of generating two-cluster dendrograms with population fractions ranging between 30% and 70% (Table 2).

**Table 2:** List of the 30 taxonomically selected two-cluster dendrograms with population fractions ranging between 30% and 70% and their corresponding IDs.

No.	Triad id:	Triad biomarkers:
1	7	protein/SH/MnSOD
2	19	protein/SHprotein/MDA
3	37	protein/TOS/MnSOD
4	50	protein/MnSOD/CuZnSOD
5	52	protein/MnSOD/MDA
6	63	SH/SHprotein/LPS
7	69	SH/CER/MnSOD
8	82	SH/TOS/MnSOD
9	91	SH/SOD/MnSOD
10	95	SH/MnSOD/CuZnSOD
11	97	SH/MnSOD/MDA
12	109	SHprotein/TAC/TOS
13	114	SHprotein/TAC/LPS
14	120	SHprotein/TOS/LPS
15	122	SHprotein/LPH/SOD
16	129	SHprotein/SOD/LPS
17	133	SHprotein/MnSOD/MDA
18	136	SHprotein/LPS/MDA
19	140	CER/TAC/MnSOD
20	146	CER/TOS/MnSOD
21	155	CER/SOD/MnSOD
22	157	CER/SOD/LPS
23	161	CER/MnSOD/MDA
24	162	CER/CuZnSOD/LPS
25	186	TOS/LPH/SOD
26	189	TOS/LPH/LPS
27	195	TOS/MnSOD/CuZnSOD
28	203	LPH/SOD/LPS
29	212	SOD/MnSOD/LPS
30	216	SOD/LPS/MDA

**Figure 1:**
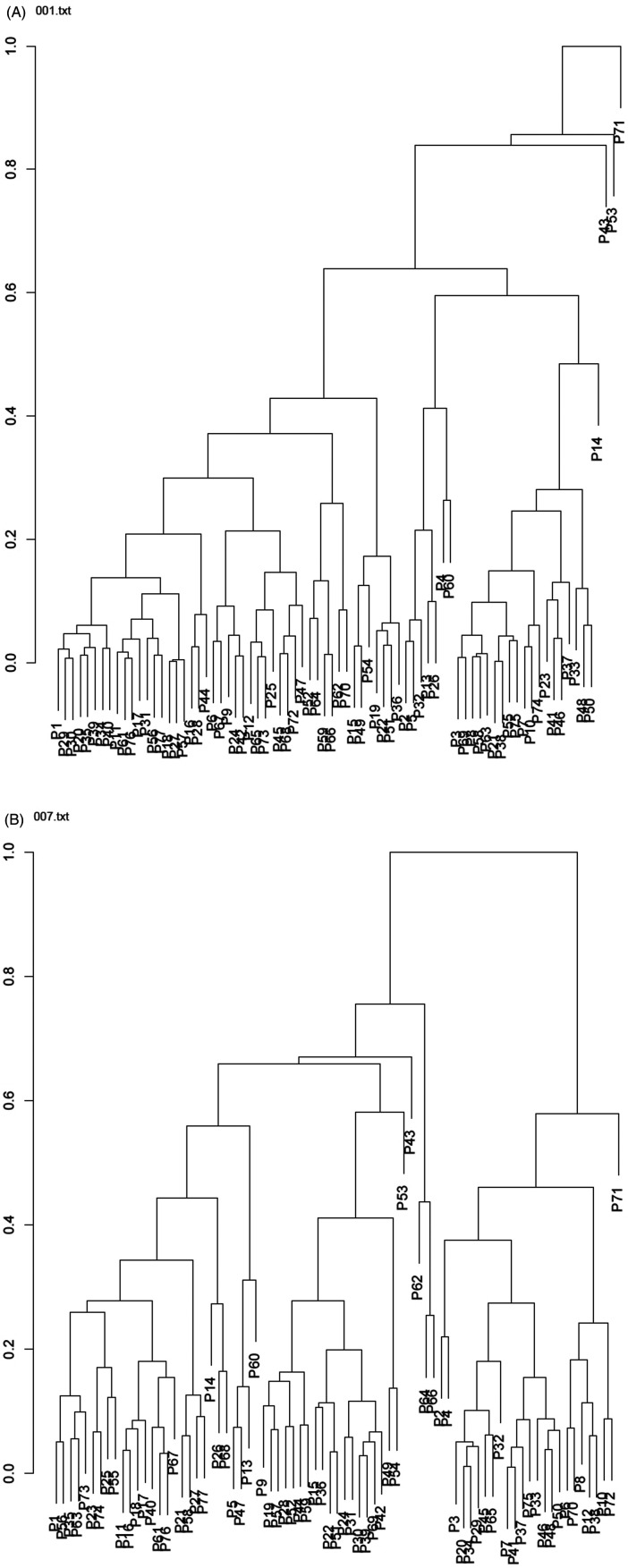
Representative examples of dendrograms obtained from triadic biomarker configurations. **(A)** Example of an unselected triad (protein–SH–SHprotein) that does not satisfy the predefined cluster balance criterion. The resulting bipartition of patients is strongly unbalanced and therefore the triad was excluded from further analysis. **(B)** Example of a selected triad (protein–SH–MnSOD) fulfilling the predefined balance condition (0.30≤ balance ≤0.70), resulting in a structurally meaningful two-cluster separation of the patient population. Dendrograms were constructed using hierarchical agglomerative clustering based on the Marczewski–Steinhaus taxonomic distance.

Representative examples of dendrograms are shown in Figure 1. An unselected dendrogram (“protein/SH/SHprotein”) and a dendrogram fulfilling the predefined criteria of two-cluster structural separation (“protein/SH/MnSOD”) are presented in Figures 1A,B, respectively.

**Figure 2:**
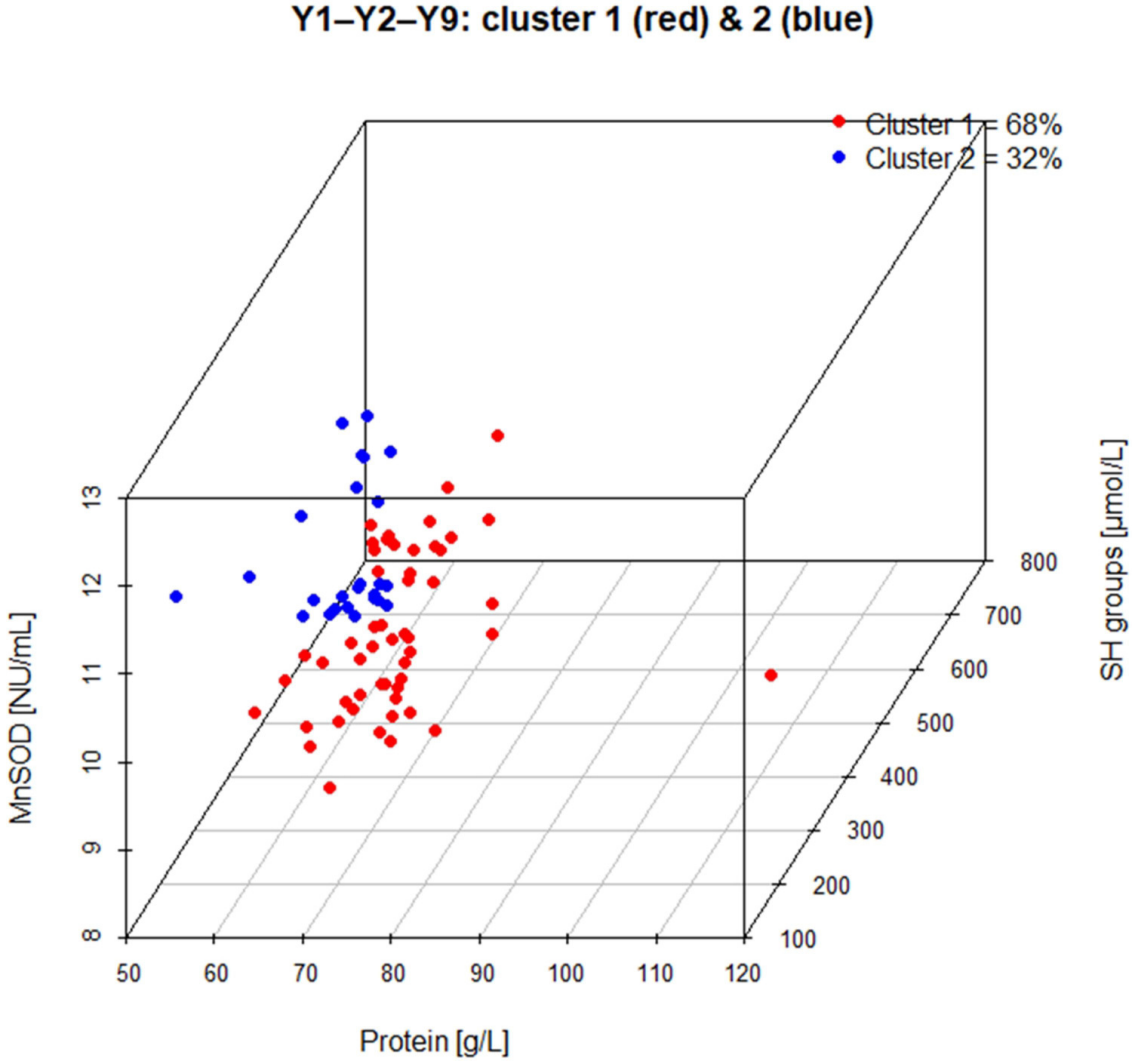
Three-dimensional representation of patient distribution for the selected biomarker triad #007 (Y1–Y2–Y9: protein, SH groups, and MnSOD). Each point represents an individual patient positioned according to the measured values of the three biomarkers. Colors indicate cluster membership derived from the corresponding dendrogram (cluster 1—red; cluster 2—blue). The clusters correspond to the top-level bipartition obtained from the hierarchical clustering analysis using the Marczewski–Steinhaus taxonomic distance.

For illustrative purposes, the assignment of patients into two clusters in the selected dendrogram for triad #007 is presented in Figure 2.

The frequency counts and percentage fractions of individual biomarkers across all 30 × 3 = 90 possible occurrences were reported in Table 3 (columns selected triads' enzyme and “n/90 fraction”). The biomarker most frequently represented among the selected 30 triads was MnSOD, appearing 16 times (corresponding to nearly one-fifth of all occurrences). The second most frequent biomarker was LPS (11 occurrences, 12%), whereas SH protein and SOD appeared 9 times each. In contrast, the least frequent biomarker contributing to the two-cluster structural separation of patients was TAC, occurring only three times among the 90 possible realizations (Table 3).

**Table 3:** Statistics of oxidative stress biomarker triads (Y1–Y12) and reproductive risk factors (X1–X10).

Code	Risk factor/enzyme	Selected triads’ biomarkers frequency	N/90 fraction	Stat. sign. (*p* < 0.05) frequency	N/30 fraction
X1	Ovarian factor	x	x	2	6.7%
X2	Tubal factor	x	x	3	10.0%
X3	Idiopathic factor	x	x	2	6.7%
X4	Endometriosis	x	x	1	3.3%
X5	Male factor	x	x	1	3.3%
X6	IVF attempt number	x	x	0	0.0%
X7	Age	x	x	1	3.3%
X8	Number of hMG/FSH administration days	x	x	0	0.0%
X9	AMH (anti-Müllerian hormone)	x	x	4	13.3%
X10	BMI (body mass index)	x	x	7	23.3%
Y1	Protein	5	5.6%	4	13.3%
Y2	Sulfhydryl (SH) groups	7	7.8%	4	13.3%
Y3	Protein-bound sulfhydryl groups (shprotein)	9	10.0%	1	3.3%
Y4	Ceruloplasmin (CER)	7	7.8%	7	23.3%
Y5	Total antioxidant capacity (TAC)	3	3.3%	3	10.0%
Y6	Total oxidant status (TOS)	8	8.9%	0	0.0%
Y7	Lipid hydroperoxides (LPH)	4	4.4%	0	0.0%
Y8	Superoxide dismutase (SOD)	9	10.0%	16	53.3%
Y9	Manganese superoxide dismutase (MnSOD)	16	17.8%	19	63.3%
Y10	Copper-zinc superoxide dismutase (CuZnSOD)	4	4.4%	17	56.7%
Y11	Lipofuscin (LPS)	11	12.2%	6	20.0%
Y12	Malondialdehyde (MDA)	7	7.8%	5	16.7%
	Total:	90	100.0%		

Counts of statistically significant results (*p* < 0.05) from Wilcoxon rank-sum and Student's *t*-tests for individual biomarkers (Y1–Y12) across the selected 30 triads, together with their percentage contributions, are summarized in the columns “stat. sign. (*p* < 0.05) frequency” and “n/30 fraction” (Table 3). IVF attempt number showed no statistically significant association with the two-cluster partitioning of patients. In contrast, BMI was the most frequently differentiating factor, reaching statistical significance seven times across the analyzed triads, i.e., in nearly one out of four triads. AMH also demonstrated a strong tendency toward cluster separation in one out of seven of the 30 triads.

The results presented in Table 3 indicate that substantially greater taxonomic differentiation occurred among the biomarkers themselves (Y1–Y12). The biomarkers most frequently associated with the two-cluster dendrogram structure were MnSOD, with 19 statistically significant outcomes (*p* < 0.05), corresponding to nearly two-thirds of the 30 selected triads. This was followed by CuZnSOD (17 occurrences) and SOD (16 occurrences), both of which were associated with cluster separation in more than half of the analyzed triadic configurations. Such pronounced taxonomic differentiation indicates an increased geometric and clinical dynamism of SOD biomarkers within the entire set of oxidative stress enzymes analyzed (Y1–Y12). In contrast, SH protein showed the lowest level of statistical differentiation across clusters, indicating the greatest similarity to the remaining biomarkers.

The results of Student's *t*-tests (together with mean values and mean differences) for individual clinical response variables (Y), comparing clusters 1 and 2 across all (2 + 3 + 2 + 1 + 1 + 1 + 4 + 7=) 21 triads that were significantly associated (*p* < 0.05) with risk factors (X) (*p* < 0.05; Table 3), were presented in Table 4.

**Table 4:** Cluster-stratified comparisons of clinical risk factors (X, panel A) and biomarker-level structural differences (Y, panel B) including mean values, mean differences, and test statistics across all 21 statistically significant triads (*p* < 0.05).

PANEL:	A					B					
Risk factor (X)	Triad ID	Mean(cluster1)	Mean(cluster2)	Mean diff.	*P*-value	Biomarker (Y)	Mean(cluster1)	Mean(cluster2)	Mean diff.	*P*-value	Stat. sign. (*p* < 0.05)
Ovarian factor	146	37%	9%	−28%	0.0055	CER	37.92	31.94	−5.98	0.0053	+
	146					TOS	11.70	10.34	−1.36	0.2328	−
	146					MnSOD	9.84	11.13	1.29	0.0000	+
	157	38%	16%	−22%	0.0345	CER	41.0	32.1	−8.90	0.0011	+
	157					SOD	18.93	20.67	1.74	0.0000	+
	157					LPS	317.8	336.4	18.6	0.3768	−
Tubal factor	120	3%	22%	19%	0.0250	SHprotein	4.52	4.58	0.06	0.7673	−
	120					TOS	11.12	11.01	−0.11	0.9233	−
	120					LPS	412.3	274.3	−138.0	0.0000	+
	129	0%	22%	22%	0.0096	SHprotein	4.66	4.51	−0.15	0.4864	−
	129					SOD	19.9	20.12	0.22	0.4746	−
	129					LPS	421.8	280.2	−141.6	0.0000	+
	189	7%	24%	17%	0.0399	TOS	11.24	10.82	−0.42	0.7225	−
	189					LPH	7.00	6.94	−0.06	0.9291	−
	189					LPS	390.2	253.5	−136.7	0.0000	+
Idiopathic factor	109	20%	42%	22%	0.0464	SHprotein	4.70	4.39	−0.31	0.1008	−
	109					TAC	1.18	1.06	−0.12	0.0000	+
	109					TOS	10.22	12.31	2.09	0.1324	−
	114	20%	50%	30%	0.0076	SHprotein	4.63	4.44	−0.19	0.2719	−
	114					TAC	1.17	1.05	−0.12	0.0000	+
	114					LPS	339.9	308.9	−31.0	0.1051	−
Endometriosis	19	0%	12%	12%	0.0460	protein	69.00	71.36	2.36	0.1379	−
	19					SHprotein	4.56	4.58	0.02	0.9437	−
	19					MDA	0.72	1.21	0.49	0.0000	+
Male factor	63	65%	38%	−27%	0.0174	SH	344.7	294.9	−49.8	0.0004	+
	63					SHprotein	4.83	4.32	−0.51	0.0158	+
	63					LPS	345.9	311.5	−34.4	0.1234	−
Age	63	32.0	34.6	2.6	0.0091	SH	344.7	294.9	−49.8	0.0004	+
	63					SHprotein	4.83	4.32	−0.51	0.0158	+
	63					LPS	345.9	311.5	−34.4	0.1234	−
AMH	19	4.31	2.86	−1.45	0.0315	protein	69.00	71.36	2.36	0.1379	−
	19					SHprotein	4.56	4.58	0.02	0.9437	−
	19					MDA	0.72	1.21	0.49	0.0000	+
	52	4.52	2.93	−1.59	0.0285	protein	68.48	71.11	2.63	0.0835	−
	52					MnSOD	10.62	10.28	−0.34	0.1287	−
	52					MDA	0.70	1.16	0.46	0.0000	+
	97	3.90	2.56	−1.34	0.0235	SH	310.5	337.5	27.0	0.1918	−
	97					MnSOD	10.8	9.44	−1.36	0.0000	+
	97					MDA	0.95	1.13	0.18	0.0270	+
	161	2.65	3.98	1.33	0.0339	CER	37.91	33.55	−4.36	0.0523	−
	161					MnSOD	9.76	10.79	1.03	0.0000	+
	161					MDA	1.18	0.90	−0.28	0.0003	+
BMI	109	23.6	21.2	−2.4	0.0007	SHprotein	4.70	4.39	−0.31	0.1008	−
	109					TAC	1.18	1.06	−0.12	0.0000	+
	109					TOS	10.22	12.31	2.09	0.1324	−
	114	23.3	21.1	−2.2	0.0022	SHprotein	4.63	4.44	−0.19	0.2719	−
	114					TAC	1.17	1.05	−0.12	0.0000	+
	114					LPS	339.9	308.9	−31.0	0.1051	−
	155	23.6	21.7	−1.9	0.0211	CER	37.0	32.2	−4.80	0.0207	+
	155					SOD	19.38	20.83	1.45	0.0000	+
	155					MnSOD	9.96	10.87	0.91	0.0000	+
	162	23.2	21.7	−1.5	0.0368	CER	37.9	29.0	−8.90	0.0000	+
	162					CuZnSOD	9.43	10.11	0.68	0.0105	+
	162					LPS	366.3	259.6	−106.7	0.0000	+
	186	23.4	21.7	−1.7	0.0237	TOS	11.32	10.71	−0.61	0.5934	−
	186					LPH	7.18	6.68	−0.50	0.4372	−
	186					SOD	19.33	21.12	1.79	0.0000	+
	203	23.4	21.8	−1.6	0.0446	LPH	7.32	6.54	−0.78	0.2283	-
	203					SOD	19.31	21.04	1.73	0.0000	+
	203					LPS	319.1	333.9	14.8	0.5372	−
	212	23.7	21.7	−2.0	0.0220	SOD	19.27	20.81	1.54	0.0000	+
	212					MnSOD	9.79	10.95	1.16	0.0000	+
	212					LPS	309.5	339.8	30.3	0.1570	−

As shown in Table 4 (Panel B), the ovarian factor was associated with changes in CER and MnSOD concentrations within structural triad #146 (*p* < 0.05, “+”). A decrease in the fraction of the study population representing this risk factor from 37% to 9% (Panel A) resulted in a reduction of CER concentration from 38 units to 32 (Panel B), indicating a direct relationship between the ovarian factor and CER levels. In contrast, the same proportional decrease in this risk factor (triad #146, Panel A) was statistically associated with an increase in MnSOD concentration from 9.8 to 11.1 (Panel B), reflecting an inverse relationship between ovarian factor and MnSOD.

An increased prevalence of the tubal factor was associated with a decrease in LPS levels across triads #120, #129, and #189, while a greater involvement of the idiopathic factor corresponded to lower TAC levels (triads #109 and #114).

Similarly, endometriosis (triad #019) was associated with increased MDA levels. A reduction in the proportion of the male factor (from 65% to 38%) and an increase in patient age (triad #63) independently resulted in decreases of approximately 50 units in SH concentrations and 0.5 units in SH protein levels. In contrast, increased AMH concentration was predominantly associated with lower MDA levels across all four triadic configurations (#019, #052, #097, and #161).

The strongest relationship was observed for BMI, which most frequently showed statistically significant associations (*p* < 0.05, “+”) with the SOD biomarker. In four of the seven relevant triads (#155, #186, #203, and #212), a decrease in BMI (1.9, 1.7, 1.6, and 2.0 kg/m^2^, respectively) was associated with an increase in SOD levels by 1.5, 1.8, 1.7, and 1.5 units, indicating an inverse relationship between BMI and SOD concentrations (Table 4).

**Figure 3:**
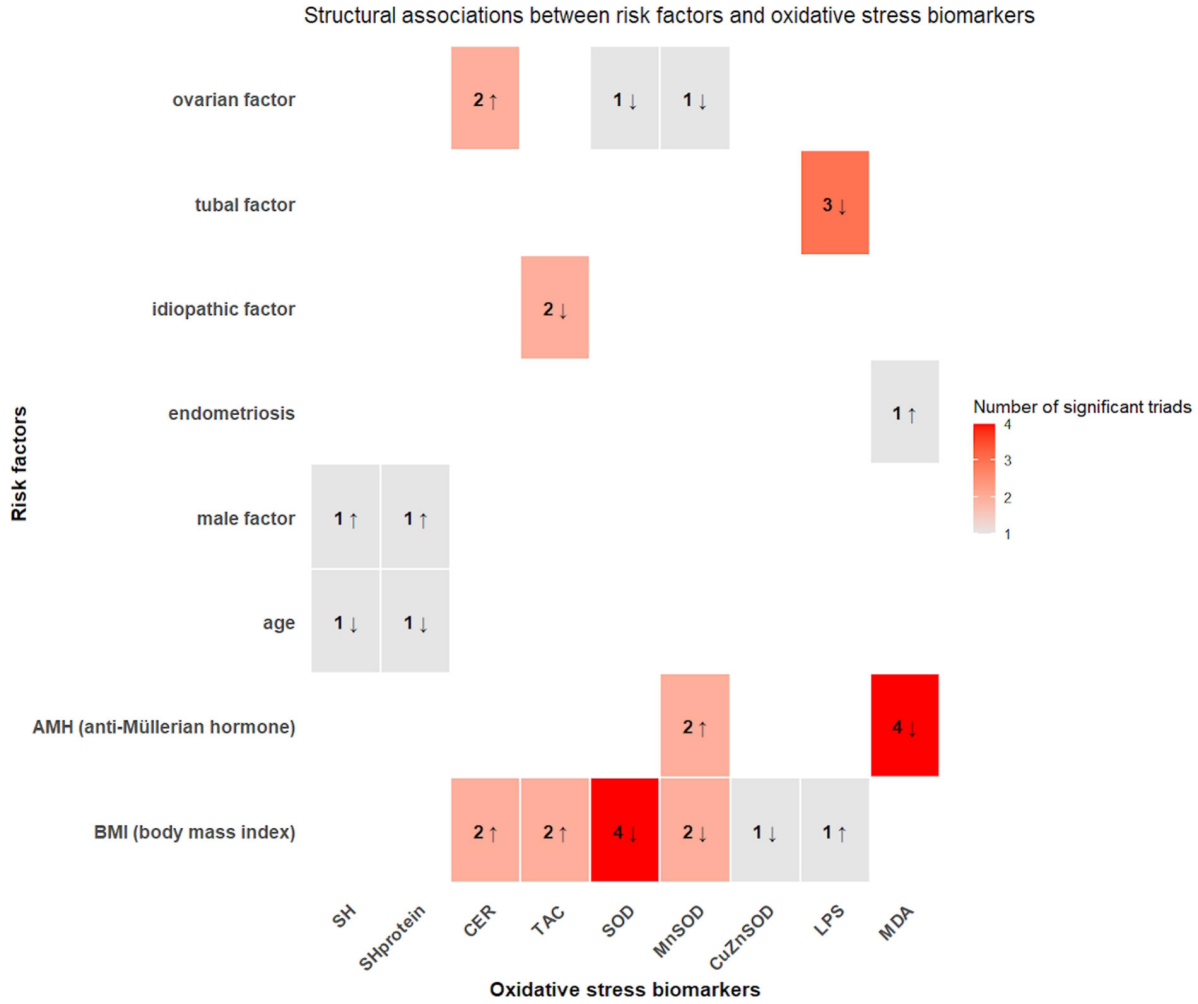
Conceptual heatmap summarizing the structural associations between clinical risk factors and oxidative stress biomarkers. The numbers in each cell indicate the number of statistically significant triads linking a given risk factor with a specific biomarker, while arrows denote the direction of the relationship (↑ direct proportionality, ↓ inverse proportionality) inferred from the mean differences reported in Table 4.

A conceptual heatmap summarizing these relationships is presented in Figure 3, providing a visual complement to the results reported in Table 4.

## 4 Discussion

Infertility is a common condition with far-reaching consequences for individuals, couples, and society. Among the modifiable lifestyle factors influencing reproductive health, body mass index (BMI) is one of the most important determinants. Numerous studies indicate that deviations from the normal BMI range—both toward underweight and overweight/obesity—adversely affect female fertility. Zhu et al. demonstrated an U-shaped association between BMI and infertility risk, indicating an increased reproductive risk at both extremes of adiposity (33). Multiple biological mechanisms underpin these observations. Underweight women often present with nutritional deficiencies, including insufficient intake of dietary antioxidants, proteins, and micronutrients such as selenium, zinc, and vitamins C and E—cofactors essential for maintaining antioxidant enzyme activity and overall redox balance (34). In contrast, higher body mass index (BMI) has been positively associated with infertility risk, with a 6% increase in prevalence observed per unit rise in BMI (OR = 1.06, 95% CI: 1.03–1.09; *P* < 0.001). Age-stratified analyses further indicated a stronger mediating contribution of BMI among younger women, accounting for 9.8% of the total effect in those aged 20–30 years and 9.96% in women aged 30–40 years (35).

The present study was not designed to evaluate protocol-specific effects of ovarian stimulation on oxidative stress biomarkers. Both GnRH agonist and antagonist protocols were therefore analyzed within a single cohort, reflecting real-world clinical practice. Importantly, all samples were collected at a standardized time point (on the day of ovulation triggering), which minimizes variability related to treatment timing. In this context, stimulation protocol was treated as a shared clinical background characteristic rather than a primary stratification variable, and the analysis focused on identifying structural relationships between biomarkers and clinical factors.

### 4.1 BMI and SOD with isoenzymes

In our study, the BMI of the enrolled patients ranged from 17.4–36.3 kg/m^2^ (mean 22.7 ± 3.5 kg/m^2^, median 21.8 kg/m^2^). The analysis of 220 variable combinations enabled the construction of a model that illustrates the relationships among multiple clinical outcomes and identifies those exerting the strongest influence within each triad. The results clearly demonstrate that BMI, more than any other assessed risk factor, has the greatest impact on disturbances in redox homeostasis. With increasing BMI, particularly in the overweight and obese range, a progressive shift toward oxidative imbalance is observed.

Obesity is characterized by chronic low-grade inflammation, driven by hypertrophic adipocytes—especially within visceral fat depots—which secrete pro-inflammatory cytokines such as TNF-α, IL-6, and leptin (11). These cytokines activate macrophages and promote the generation of reactive oxygen species (ROS). In parallel, mitochondrial dysfunction develops, resulting in impaired oxidative phosphorylation and increased leakage of free radicals from the electron transport chain Pan et al. (36). This pro-oxidative state is aggravated by a decline in antioxidant defences, as obesity is associated with reduced activity of key enzymes, including superoxide dismutase (SOD), catalase (CAT), and glutathione peroxidase (GPx), as well as decreased glutathione (GSH) availability. Among the analyzed biomarkers, MnSOD appeared most frequently in the cluster-separating triads (19 occurrences), followed by CuZnSOD (17 occurrences) and total SOD (16 occurrences). These findings suggest that SOD isoenzymes are among the most dynamic components of the antioxidant system in women undergoing infertility treatment. In contrast, SH protein showed the lowest level of statistical differentiation across clusters, indicating the greatest similarity to the remaining biomarkers.

The depletion of antioxidant defence mechanisms—including superoxide dismutase (SOD) and its isoenzymes—leads to the accumulation of reactive oxygen species (ROS). Excess ROS initiates lipid peroxidation through radical chain reactions targeting polyunsaturated fatty acids in cellular membranes, resulting in loss of membrane integrity, altered fluidity, and impaired ion transport (21).

### 4.2 BMI and ceruloplasmin

Beyond its effects on SOD and its isoenzymes, BMI also significantly influenced ceruloplasmin (CER) levels. We observed that decreasing BMI was associated with a reduction in serum ceruloplasmin (CER), an effect particularly evident among women whose infertility was attributed to ovarian factors. Although low CER levels are classically recognized as a hallmark of Wilson disease, emerging evidence suggests a broader relevance of CER in reproductive biology (37, 38). Ceruloplasmin is highly vulnerable to reactive oxygen species, and oxidative modification of the protein results in loss of structural stability, release of its bound copper ions, and impairment of its ferroxidase function. Using proteomic profiling, Lee et al. identified CER—alongside four other reproductive system–related proteins—as a potential biomarker for primary ovarian insufficiency (37). Under conditions of pronounced redox imbalance, such as diabetes or chronic inflammatory states, ceruloplasmin may shift from an antioxidant molecule toward a protein exhibiting pro-oxidant properties (39). In addition to its established ferroxidase activity and antioxidant function, CER modulates inflammatory and oxidative pathways implicated in obesity. Emerging evidence also suggests that CER may influence metabolic status through interactions with the intestinal microbiota and may contribute to obesity-related systemic complications. These properties position CER as a potentially meaningful link between metabolic health, oxidative stress, and female reproductive function (18, 37, 38, 40).

### 4.3 BMI and TAC

Another observation in our study was a decline in TAC with decreasing BMI. Within the SH-protein/TAC/TOS triad, decreasing BMI was associated with a statistically significant reduction in TAC, while TOS showed an increasing trend; however, this increase did not reach statistical significance (*p* = 0.132).

Changes in TAC were also noted in patients diagnosed with idiopathic infertility. Total antioxidant capacity (TAC) reflects the integrated effect of all antioxidants present in plasma, including uric acid—one of the strongest endogenous plasma antioxidants—bilirubin, fat-soluble vitamins such as vitamins A and E, albumin, and various dietary phenolic compounds (41). Consequently, TAC is influenced not only by body weight but also by metabolic status and dietary intake.

Previous studies have reported reduced TAC levels in individuals with obesity across both pediatric and adult populations, suggesting that this phenomenon is independent of age (42). Uric acid plays a central role in determining plasma antioxidant potential, contributing approximately 67% of total antioxidant capacity (43). However, uric acid exhibits both antioxidant and pro-oxidant properties depending on its concentration and the surrounding biochemical environment (44). It has been described as a “double-edged sword” because its biological activity differs between physiological and elevated concentrations and may influence processes such as endothelial cell aging. Because uric acid is unstable at near-neutral or acidic pH, it can undergo auto-oxidation, generating hydrogen peroxide. This instability may promote the consumption of endogenous antioxidants and ultimately alter systemic antioxidant status (43).

These mechanisms may help explain our findings, in which TAC decreased with decreasing BMI—contrary to the common expectation that TAC declines with higher BMI—suggesting that variations in uric acid turnover and metabolic status may differentially influence systemic antioxidant capacity across BMI categories.

### 4.4 BMI and MDA

High BMI is associated with persistent oxidative stress and chronic low-grade inflammation, which promote enhanced oxidative reactivity and impairment of antioxidant defence systems (45). In our cohort, higher BMI correlated with decreased SOD activity and TAC levels, accompanied by increased MDA concentrations, indicating a predominance of lipid peroxidation processes over the protective capacity of antioxidant systems.

The high reactivity of MDA is primarily due to its electrophilicity, which enables interactions with nucleophilic targets such as lysine, histidine, and arginine residues in proteins. These reactions lead to the formation of Schiff-base adducts, classified as advanced lipid peroxidation end products (21). Under conditions of oxidative stress, further metabolism of MDA promotes the generation of malondialdehyde–acetaldehyde adducts, which exhibit strong immunogenic properties (46). MDA-derived adducts can participate in secondary deleterious reactions, including intra- and intermolecular protein and DNA crosslinking, resulting in profound alterations of biomolecular structure and function and their accumulation during aging and chronic disease. MDA is also a significant contributor to DNA damage and mutagenesis (47, 48).

A decrease in SOD activity among our patients, accompanied by elevated MDA levels, reflects intensified oxidative stress within the studied cohort. In obesity-associated models, increased free fatty acid availability enhances lipid peroxidation, promoting MDA accumulation and oxidative damage in tissues.

Additionally, our observations showed increased MDA levels in patients diagnosed with endometriosis. Endometriosis is characterized by profound immune dysregulation and chronic inflammatory activation, which are closely linked to excessive production of reactive oxygen species, impaired antioxidant defences, and enhanced lipid peroxidation (49). Our findings are consistent with previous reports demonstrating elevated oxidative stress markers in women with endometriosis (50), Rambulangi & Ardianta Widyanugraha (51),. However, some studies suggest an inverse association between BMI and endometriosis, indicating a lower prevalence of the disease among women with higher BMI (52), Dai et al. (49). Consequently, the etiological contribution of BMI to endometriosis remains uncertain, which may explain the relatively weak associations observed in our dataset between the endometriosis factor and other analyzed clinical or biochemical variables (53).

In the present study, we also identified a significant association between malondialdehyde (MDA) and anti-Müllerian hormone (AMH) levels. Serum levels of AMH, as a marker of ovarian function, are influenced by various genetic and environmental factors—including obesity (54, 55). Specifically in our patients, decreasing AMH concentrations were accompanied by a corresponding increase in MDA levels. Both in cases of low AMH resulting from advanced reproductive age and in diminished ovarian reserve due to other reasons, higher MDA levels may be explained by more intense lipid peroxidation processes affecting cellular membranes F. Yan et al. (8).In the studied cohort, these processes may also be interdependent on adipose tissue mass and the efficiency of antioxidant defence systems. Nevertheless, our observations indicate that elevated BMI exerts a more detrimental effect on redox homeostasis than extreme AMH values, whether low or high. We demonstrated a clear trend in SOD and MDA dynamics, with increasing AMH levels being associated with higher MnSOD activity and lower MDA concentrations. In contrast, a declining ovarian reserve was characterized by increased MDA levels accompanied by a parallel reduction in MnSOD.

### 4.5 BMI and lipofuscin

We observed that decreasing BMI was associated with a marked reduction in LPS levels, occurring in parallel with a concomitant decline in ceruloplasmin (CER) and an increase in CuZnSOD activity. Lipofuscin, a product of oxidative degradation of lipoproteins and proteins, accumulates in long-lived cells under conditions of chronic oxidative stress and may reflect cumulative oxidative damage in reproductive tissues. Experimental studies have demonstrated increased lipofuscin accumulation in ovarian tissue of obese animal models, accompanied by markers of cellular aging and inflammatory infiltration (56).

Consistent with these observations, our findings indicate that lower BMI is associated with reduced lipofuscin levels, suggesting that, conversely, higher BMI may promote a cellular environment characterized by accelerated aging. Lipofuscin accumulation may contribute to mitochondrial dysfunction and impaired autophagy in ovarian cells, potentially leading to reduced oocyte quality and diminished ovarian reserve in obesity. Considering lipofuscin as a marker of long-term oxidative stress may therefore provide additional insight into the mechanisms linking BMI, chronic oxidative burden, and reproductive dysfunction. This interpretation is supported by the disturbances observed in the redox balance of the studied cohort, particularly within the SOD–MDA–TAC axis of oxidative stress regulation (57).

These findings should be interpreted in the context of a clinically heterogeneous IVF population in which different stimulation protocols were analyzed jointly rather than comparatively.

### 4.6 Limitations

The present study has several limitations. First, the relatively small cohort size may limit the statistical stability of complex multidimensional analyses; therefore, the results, are best regarded as hypothesis-generating rather than confirmatory. In addition, the cross-sectional design of the study does not allow for the assessment of temporal relationships or causal inference between clinical risk factors and oxidative stress biomarkers.

1. Body mass index (BMI), while widely used, represents total body mass and does not distinguish between lean mass, bone mass, or the relative contribution of visceral vs. subcutaneous adipose tissue, which are biologically distinct in their metabolic and oxidative profiles. Relative fat mass (RFM) has been proposed as a more accurate surrogate of adiposity; however, its relevance to female infertility and redox homeostasis remains insufficiently explored (58). Recent consensus reviews indicate that BMI is a practical and useful tool for population-level surveillance and primary care screening, yet its ability to predict chronic disease risk and excess adiposity at the individual level is limited. Importantly, BMI was originally derived from a homogeneous cohort of White, middle-aged European men, which constrains its generalizability across sex, age, and ethnic groups. Moreover, BMI does not capture fat distribution and therefore fails to account for metabolically detrimental central adiposity, while also risking misclassification in individuals with higher muscle mass or larger skeletal frames (59, 60). Recent studies have suggested that alternative anthropometric indices, such as the Body Roundness Index (BRI) and A Body Shape Index (ABSI), may better reflect visceral adiposity and metabolic risk and have been associated with outcomes of assisted reproductive technologies (61). Alternative anthropometric and body composition measures may better overcome the limitations of BMI. Body fat percentage provides a more direct assessment of adiposity, while indices of fat distribution, such as waist-to-hip ratio and waist circumference, more accurately reflect metabolically relevant abdominal fat and cardiometabolic risk. Moreover, evidence supporting the “fat but fit” paradigm indicates that physical fitness modifies the relationship between adiposity and health outcomes, underscoring that BMI alone is insufficient to capture the interplay between body composition, metabolic fitness, and clinical risk (62). In our study, questionnaire-based data on lifestyle factors among women undergoing infertility treatment were unavailable, and no additional assessments of body fat content or distribution were performed. Furthermore, we were unable to account for physical activity levels, including exercise intensity and frequency, in the analyzed cohort. Consequently, while BMI remains a convenient proxy of adiposity, its limitations—particularly regarding fat distribution and metabolic heterogeneity—should be acknowledged when interpreting associations between body weight, oxidative stress, and antioxidant defence in women undergoing infertility treatment.
2. In infertility treatment, a growing reliance on dietary supplements and so-called add-on therapies has emerged (63, 64). While some of these interventions may support selected aspects of treatment, they do not comprehensively address systemic metabolic regulation. Adipose tissue constitutes the body's largest endocrine organ, and both its excess and deficiency—according to an extensive body of evidence—adversely affect immune defence mechanisms and normal physiological processes (11). It is therefore plausible that, in a subset of patients, optimizing body weight and the proportions and distribution of adipose tissue could improve IVF outcomes or, in some cases, reduce the need for assisted reproductive technologies altogether.
3. In the present study, we did not assess additional factors that may potentially modulate the systemic response to chronic inflammation associated with increased BMI. It is plausible that an analysis incorporating a broader range of variables beyond the twelve considered here would reveal further associations; however, such an approach would necessarily require a substantially larger study cohort. In addition, we did not stratify the analysis according to the type of ovarian stimulation protocol (GnRH agonist vs. antagonist). Controlled ovarian hyperstimulation itself may influence systemic oxidative stress parameters through increased reactive oxygen species (ROS) production. Although both protocols are routinely used in clinical practice and reflect real-world IVF populations, and available literature provides inconsistent evidence regarding protocol-specific effects on oxidative stress markers, we cannot exclude a potential modulatory influence of stimulation protocol on the observed biomarker profiles (65–67). Within the applied triad-based framework, stimulation protocol was treated as a shared clinical background characteristic rather than a primary stratification variable. Future studies incorporating protocol-stratified analyses and pre-stimulation baseline measurements would allow for a more precise evaluation of the impact of ovarian stimulation on redox homeostasis.

## 5 Conclusion

The statistical modeling approach applied in our study enabled the analysis of structural relationships between infertility risk factors and oxidative stress–related enzymes and biomarkers, providing an alternative to classical regression-based methods for exploring multidimensional clinical data.

Our findings indicate that BMI is one of the key modifiable factors associated with disturbances in redox homeostasis in women undergoing infertility treatment, with antioxidant enzymes playing a central role in these relationships. With increasing BMI—particularly in the overweight and obese range—a shift toward a pro-oxidative milieu was observed, manifested by decreased superoxide dismutase (SOD) activity and alterations in its isoenzymes, along with intensified lipid peroxidation (increased MDA) and reduced total antioxidant capacity (TAC). Such biomarker profiles suggest that enzymatic antioxidant defence is among the most dynamic and clinically sensitive components of the redox system in women with reproductive disorders.

SOD-related biomarkers—especially MnSOD and CuZnSOD—showed particularly strong relevance, as they most frequently contributed to the cluster-separating structure and differentiated triadic configurations. This may indicate that mitochondria (where MnSOD predominates) constitute a critical site of oxidative stress generation in the context of increased body mass, while also representing a compartment in which adaptive antioxidant mechanisms become overwhelmed. Reduced activity of SOD and its isoenzymes promotes ROS accumulation, triggering a cascade of lipid peroxidation, membrane destabilization, and disruption of cellular functions essential for oocyte maturation and embryo developmental competence.

In parallel, ceruloplasmin (CER) exhibited BMI-related variation, suggesting that redox-dependent alterations in metal-transport proteins (particularly those involved in copper and iron handling) may further modulate oxidative balance. Changes in TAC highlight the integrated role of both enzymatic and non-enzymatic antioxidant systems in maintaining systemic redox equilibrium.

Taken together, these findings support the concept that impaired antioxidant enzyme activity—primarily involving SOD and its isoenzymes—constitutes an important mechanistic link between abnormal body mass and female reproductive dysfunction. In this framework, redox biomarkers, particularly those related to SOD activity and lipid peroxidation (MDA), may have not only descriptive value but also prognostic and potential clinical relevance—as indicators of poorer treatment response risk and as targets for interventions aimed at restoring oxidative balance (e.g., through optimization of body weight and metabolic status).

## Data Availability

The raw data supporting the conclusions of this article will be made available by the authors, without undue reservation.
